# Evaluating the Therapeutic Effect of Sofosbuvir in Outpatients with COVID-19: A Randomized Clinical Trial Study

**DOI:** 10.31661/gmj.v13i.3035

**Published:** 2024-02-08

**Authors:** Maryam Haddadzadeh Shoushtari, Hanieh Raji, Seyed Hamid Borsi, Heshmatollah Tavakol, Bahman Cheraghian, Mahtab Moeinpour

**Affiliations:** ^1^ Air Pollution and Respiratory Diseases Research Center, Ahvaz Jundishapur University of Medical Sciences, Ahvaz, Iran; ^2^ Department of Biostatics and Public Health, Ahvaz Jundishapur University of Medical Sciences, Ahvaz, Iran; ^3^ Department of Pulmonology, School of Medicine, Ahvaz Jundishapur University of Medical Sciences, Ahvaz, Iran

**Keywords:** Sofosbuvir, COVID-19, Mild Symptoms, Mortality

## Abstract

Background: The coronavirus disease 2019 (COVID-19) pandemic has engendered scores of deaths worldwide. Just as the development of varying procedures during the pandemic has helped inhibit the disease, none is considered a definitive treatment protocol for this problem, as each induces some clinical complications pertinent to the disease. This study thus assessed the early use of sofosbuvir in outpatients with mild COVID-19. Materials and Methods: This randomized clinical trial study was conducted on 360 patients with mild COVID-19 infection at 17 Shahrivar Ahvaz Health Center. These patients were randomly divided into the intervention and control groups. Both the control and intervention groups received 400 mg of sofosbuvir and a placebo for seven days, respectively. After 14 days from the onset of the treatment, the duration of symptoms, the necessity of hospitalization, the mean of hospitalization duration, and mortality were assessed. Results: The most common symptoms in the intervention and control groups were coughs with a frequency of 46 (25.6%) and 54(30%), respectively. The two groups showed no statistically significant difference in the frequency of the first observed clinical symptom related to the disease (P=0.2). The mean days that the patients were symptomatic in the control group were 14±4.17, whereas, in the intervention group, it was 12.12±3.15 (P=0.08). The frequency of hospitalization in the control and intervention groups was 7 (3.8%) and 4 (2.22%), respectively (P=0.11). Moreover, the mean days of hospitalization in the control and the intervention groups were 4±1.1 and 3±0.8, respectively (P=0.15). In addition, the two groups had a similar frequency of hospitalization in the ICU (0) and mortality rate (0). Conclusion: Sofosbuvir alone cannot play a significant role in the treatment of outpatients with mild COVID-19.

## Introduction

Since the severe acute respiratory syndrome-coronavirus-2 (SARS-CoV-2) first appeared, it caused a serious threat to the general public’s health [1]. As shown, the majority of SARS-CoV-2 infected patients exhibit no symptoms or rather mild symptoms, necessitating outpatient care. Only a small percentage of individuals progress to develop serious diseases to the point that they mostly necessitate hospitalization [2]. The impact of coronavirus disease 2019 (COVID-19) on the healthcare system and morbidity and mortality can both be decreased by stopping the disease’s course.

To present, several alternative therapies have been created and are being used to treat non-hospitalized individuals with mild to moderate COVID-19 to prevent the progression of the disease. The most effective treatment for SARS-CoV-2 infection is still unknown despite numerous studies on treatment regimens for the management of COVID-19 patients [3][4]. The coronaviruses are single-stranded RNA viruses that share characteristics in common with hepatitis C virus (HCV) and other single-stranded RNA viruses. RNA-dependent RNA polymerase (RdRp) is required for the replication process of certain positive-sense single-strand RNA viruses, including coronaviruses like HCV and flaviviruses [5][6]. RdRp inhibitors, which are used to treat HCV, may also work to treat SARS-CoV-2, according to a popular theory. A different medication is used as a COVID-19 therapeutic protocol because sofosbuvir is known to be a well-tolerated and effective direct-acting antiviral (DAA) against HCV [7] sofosbuvir-terminated RNA was much more resistant to exonuclease rejection than remdesivir-terminated RNA [8].

Given that sofosbuvir is available in Iran and is both cost-effective and safe [9], our goal was to assess sofosbuvir’s effectiveness exclusively in patients with mild COVID-19. So, we did not analyze it in combination with any other medications.

## Materials and Methods

**Figure-1:**
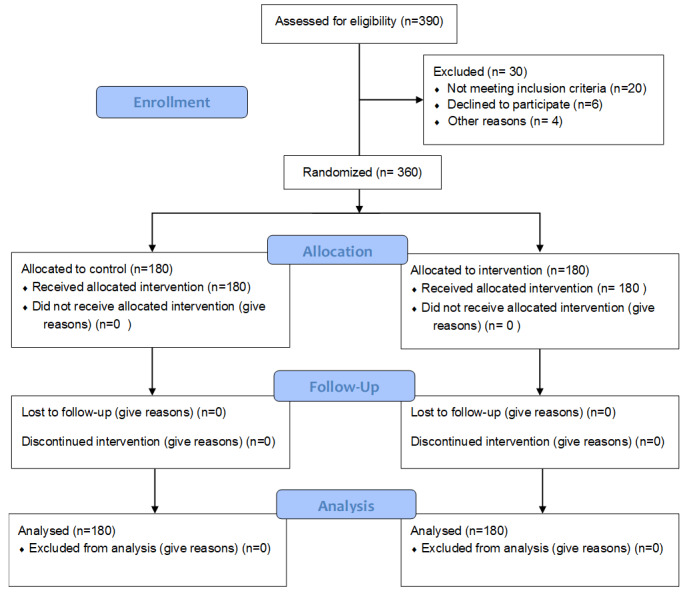
Figure 1. Grouping and random allocation of the patients into two control and intervention groups based on the entry and exit criteria

**Table:** Table1. Comparison of the Baseline Characteristics between the Groups

**Variables**	**Control group**	**Intervention group **	**P-value**
**Age (year)**	37.76±11.6	39.1±14.41	0.2
**History of cigarette and waterpipe smoking**	13(7.1 %)	19(10.5%)	0.49
**Gender** **Men** **Women**	56 (31.1%) 124 (68.9%)	106 (58.9%) 74 (41.1%)	0.075
**Weight**	80.58±22.5	80.44±16.89	0.94
**Height**	171.31±9.8	173.54±7.89	0.1

This randomized clinical trial study was conducted for 3 months from October 23, 2021, to January 21, 2022, at 17 Shahrivar Ahvaz Health Center, and each patient was followed up for 14 days after initiation of the intervention. Outpatients with positive PCR and mild symptoms (mild symptoms include sore throat, dry cough, vomiting, body pain, nausea, oxygen saturation percentage greater than or equal to 93, fever less than 38 degrees, as well as stable blood pressure and pulse) were eligible to participate. In addition, individuals with the age less than 18 years, history of organ transplantation, history of severe skin diseases, history of sensitivity and allergy to the studied drug, previous treatment with the studied drug, inability to swallow nine pills, glucose-6-phosphate dehydrogenase (G6PD) enzyme deficiency, experienced treatment with anticonvulsant drugs, pregnancy or breastfeeding, advanced liver, lung, heart, and hematological disease, having tumor as well as neurological diseases including CVA were excluded. In this study, 390 patients were enrolled, of which 30 were excluded based on the exclusion criteria, and finally, 360 patients affected with mild COVID-19 infection with PCR (+) test who had been referred to the outpatient treatment centers in Ahvaz were included in the study. These patients were randomly divided into two groups: the intervention group (under usual treatment + sofosbuvir (400 mg) (Aburaihan Pharmaceutical Company) as one pill daily for 7 days) and the control group (under usual treatment + placebo as one tablet daily for 7 days).

The random block method was used to assign patients to the study groups. To avoid information bias, this study was designed as a triple blind. In this way, the patients were unaware of the allocation. Also, the person assessing the treatment result was unaware of the type of treatment.

For minimizing the bias in the study, the allocation concealment method was used. Seven days after finishing the medicine (after 14 days from the onset of the treatment), the results of the mean number of days for symptoms, frequency of the need to be admitted to the ward/ intensive care unit (ICU), and frequency of the mortality of the two groups were assessed. Figure-1 illustrates the allocation and grouping of patients.

Statistical Analysis

Data was analyzed through SPSS (version 22, IBM Crop., Armonk, NY, USA). Classified variables were expressed as numbers and percentages. Chi-square and independent t-test were used to compare the data of the two groups. In addition, Fisher’s exact test was applied to data dispersion. P<0.05 was considered significant.

Ethical Considerations

This study was conducted after receiving the approval of the Ethics Committee of Ahvaz University of Medical Sciences (IR.AJUMS.REC.1399.754). The study was also registered in the Iran Clinical Trial Database (IRCT) (IRCT ID: IRCT20201127049505N1; https://fa.irct.ir/trial/53212). Written informed consent was signed by all the patients or their companions for participating in the study.

## Results

In this study, 360 patients with mild COVID-19 symptoms and positive PCRs participated. All participants were allocated evenly and randomly into two intervention and control groups. Table-1 provides a summary of the patient baseline characteristics for the two groups.

Table-1 demonstrates that there were no statistically significant differences in age, gender, and history of cigarette and water pipe smoking between the two groups (P>0.05).

The frequency of the first recognized clinical sign of the disease (cough, fever, headache, weakness, sore throat, and myalgia) was also compared between the two groups (Table-2).

The frequency of cough was 46 (25.6%) and 54 (30%) in both groups, and there were no statistically significant differences between the two groups for any of the other variables, including fever, headache, weakness, sore throat, and myalgia (P=0.2).

Table-3 displays the clinical result, including the mean days that symptom persisted, the mean days that patients were hospitalized, and the frequency of hospitalization in the two groups. According to Table-3, the mean number of days the patients were symptomatic in the control group was 144.17, but it was 12.123.15 in the intervention group (P=0.08). Hospitalization rates were 7 (3.8%) and 4 (2.22%), respectively, in the control and intervention groups (P=0.11). In addition, the control and intervention groups’ respective mean hospitalization durations were 41.1 and 30.8 days (P=0.15).

As this Table shows, even if some variables in the intervention group exhibited a more favorable ratio, the observed difference was not statistically significant (P>0.05).

Additionally, there was no difference in the mortality rate and the frequency of ICU hospitalizations (both 0).

In terms of the frequency of drug-related side effects, including nausea and vomiting, diarrhea, and headache, there was no statistically significant difference between the intervention group and the control group, and the symptoms did not disqualify any participants from the study (P<0.05).

## Discussion

**Table:** Table2. Comparison of the Frequency of the First Presented Clinical Symptom between the Two Groups

Variable	Control	Intervention	P-value
Cough	54(30%)	46(25.6%)	
Headache	34(18.9%)	29(16.1%)	
Sore throat	8(4.4%)	15(8.3%)	0.2
Weakness	35 (19.4%)	37 (20.6%)	
Myalgia	7 (3.9%)	8 (4.4%)	
Fever	2 (1.1%)	3 (1.7%)	

**Table:** Table3. Comparison of Clinical Outcome between the Two Groups

Variables	Control	Intervention	P-value
Mean days of the presence of symptoms	14±4.7	12.12±3.15	0.08
Mean days of hospitalization	4±1.1	3±0.8	0.15
Frequency of the need for hospitalization	7 (3.8%)	4 (2.2%)	0.11

The goal of this study was to study the effect of sofosbuvir as a treatment for decreasing symptom duration, hospital stays, ICU stays, and COVID-19-related deaths. According to the findings, there was no discernible difference in the course of the disease between the case and control groups.

Nourian et al. assessed the impact of sofosbuvir in the treatment of COVID-19 and based on the similarity between the replication mechanisms of HCV and coronavirus, they found that it could be a feasible option [10].

According to Sadeghi et al., the duration of the hospitalization was dramatically decreased when sofosbuvir and daclatasvir were added to standard care as opposed to standard care alone. Furthermore, there was no difference in the death rate between the two groups. Although their study involved moderate to severe hospitalized patients, the most recent findings were in line with ours, which involved mild cases of COVID-19. The length of symptom duration, frequency of hospitalization, and mean days of hospitalization were not significantly different between the case and control groups, and none of our study participants required ICU admission and none passed away [11].

In contrast to our findings, sofosbuvir was found to decrease hospitalization in the study of Bozorgmehr et al. This discrepancy may be the result of various disease severity among participants; while patients in the trial by Bozorgmehr et al. had moderate COVID-19, our participants had mild COVID-19 [9]. Additionally, none of these patients required ICU care in this trial, which was consistent with our data. Assessing the impact of sofosbuvir, daclatasvir, and ribavirin on COVID-19 hospitalized patients, Abbaspour et al. found no difference between the case and control groups in terms of the frequency of ICU admissions or the frequency of fatalities [12]. The results of their study agreed with those of ours. Eslami et al. assessed the impact of sofosbuvir/daclatasvir in COVID-19 patients and found that the intervention group’s ICU stay was shorter than that of the control group. These results did not correspond to our findings [13]. The difference between the two studies could be explained by the fact that, in contrast to Islami et al.’s study, we assessed sofosbuvir’s impact on COVID-19 outpatients.

Additionally, Islami et al. assessed the impact of 600 mg of ribavirin (control group) in comparison to 600 mg of sofosbuvir and 60 mg of daclatasvir (intervention group) while we examined the impact of sofosbuvir versus placebo.

Additionally, similar to our study, Rouzbeh et al. assessed the impact of sofosbuvir and daclatasvir in the outpatient’s treatment of COVID-19 compared to the control group and found that the frequency of hospitalization was not statistically significant between the two groups [14]. Sayad et al. assessed the effectiveness of sofosbuvir/velpatasvir in patients with COVID-19 compared to standard care and found no significant difference in the time of symptom improvement and length of hospital stay compared to the control group; this study is also consistent with our findings [15]. Additionally, Simons et al. analyzed the effectiveness of sofosbuvir/daclatasvir for the treatment of COVID-19 and found that the intervention group’s rate of symptom improvement after 14 days was higher, which was inconsistent with our findings [16]. The fact that we looked at COVID-19 individuals who were outpatients rather than hospitalized may be the reason for various results. Zein et al. performed a meta-analysis on the effectiveness of sofosbuvir and daclatasvir in COVID-19 patients and found that these drugs reduced mortality and the requirement for hospitalization in intensive care units [17]. The difference between Zein’s study and ours appears to be that in Zein’s study, most of the patients had COVID-19 that was moderate to severe, and the severity in a small number of these patients was unclear, but in our study, all of the patients who had COVID-19 had mild severity.

## Conclusion

The results of our study revealed that sofosbuvir as an antiviral drug alone cannot significantly affect the recovery process of outpatients with mild COVID-19. Given that our study was performed on mild COVID-19 patients, further studies are suggested to investigate and analyze the effectiveness of the drug on patients who are in the severe phase of the disease.

## Acknowledgment

This article was extracted from the thesis prepared by Dr Mahtab Moinpour. We thank the staff of 17 Shahrivar Ahvaz Health Center and Aburaihan Pharmaceutical Company.

## Conflict of Interest

There is no conflict of interest.
